# Isolation, Characterization and Biotechnological Potentials of Thraustochytrids from Icelandic Waters

**DOI:** 10.3390/md17080449

**Published:** 2019-07-31

**Authors:** Magnús Örn Stefánsson, Sigurður Baldursson, Kristinn P. Magnússon, Arnheiður Eyþórsdóttir, Hjörleifur Einarsson

**Affiliations:** 1 School of Business and Science, University of Akureyri, Nordurslóð 2, 600 Akureyri, Iceland; 2 BioPol ltd., Einbúastíg 2, 545 Skagaströnd, Iceland; 3 Icelandic Institute of Natural History, Borgum vid Nordurslóð, 600 Akureyri, Iceland

**Keywords:** single cell oil, biomass, PUFA, docosahexaenoic acid (DHA), fish byproducts, biodiesel

## Abstract

The following study reports on the first thraustochytrid isolates identified from Iceland. They were collected from three different locations off the northern coast of the country (Location A, Skagaströnd; Location B, Hveravík; and Location C, Eyjafjörður). Using 18S rDNA sequence analysis, isolates from Locations A and B were identified within the *Thraustochytrium kinnei* species while other isolates within the *Sicyoidochytrium minutum* species when compared to other known strains. Cells isolated from Locations A (2.10±0.70 g/L) and B (1.54±0.17 g/L) produced more biomass than the ones isolated from Location C (0.43±0.02 g/L). This study offers the first-time examination of the utility of byproducts from fisheries as a nitrogen source in media formulation for thraustochytrids. Experiments showed that isolates produced more biomass (per unit of substrate) when cultured on nitrogen of marine (2.55±0.74 g/L) as compared to of commercial origin (1.06±0.57 g/L). Glycerol (2.43±0.56 g/L) was a better carbon source than glucose (1.84±0.57 g/L) in growth studies. Fatty acid (FA) profiles showed that the isolates from Location C (*S. minutum*) had low ratios of monounsaturated (4.21±2.96%) and omega-6 (0.68±0.59%) FAs. However, the isolates also had high ratios of docosahexaenoic acid (DHA; 35.65±1.73%) and total omega-3 FAs (40.39±2.39%), indicating that they could serve as a source of marine oils for human consumption and in aquaculture feeds. The *T. kinnei* isolates from Location A could be used in biodiesel production due to their high ratios of monounsaturated (18.38±6.27%) long chain (57.43±8.27%) FAs.

## 1. Introduction

Thraustochytriaceae (common name thraustochytrids) are a family of microorganisms under the Labyrinthulomycetes class (e.g., [1]). After their discovery, labyrinthulids [2] and thraustochytrids [3] were classified either as fungi or protozoa [4]. With the invention of DNA sequencing technology, Labyrinthulomycetes were placed among heterokonts [5,6]. The ongoing debate as to whether Labyrinthulomycetes are algae is ultimately a question of whether the common ancestor of Stramenopiles had plastids and subsequently lost them, or that it was never photosynthetic [7].

Despite the ongoing decline of global fisheries that began in the late 20th century [8], the major sources of omega-3 polyunsaturated fatty acids (PUFAs) remain seafood and fish oils. However, the world’s supply of these nutrients is insufficient to satisfy global demand [9,10]. PUFAs are fundamental to a balanced diet and being deficient in these nutrients can have a negative impact on health [11,12]. PUFAs are also important in aquaculture feeds, not only for the benefit of the consumer but also for the health of the cultured fish [13]. As microbes, including thraustochytrids, have been considered candidates for the commercial production of PUFAs [14,15,16], several different strains have been isolated for this purpose, and considerable efforts have been made to adjust growth conditions to optimize their omega-3 PUFA content. Most of the fats these strains synthesize are in the form of triglycerides and stored in lipid bodies within the cell; phospholipids comprise a smaller amount of the total fatty acids (e.g., [17,18,19,20]).

The global warming caused by modern anthropogenic greenhouse gas emissions has already had an impact on the world’s ecosystems [21,22]. More optimistically, a drastic reduction in emissions including carbon sequestration can minimize their impact on Earth’s biota [23]. For this to manifest, an alternative fuel source to fossil fuels will need to be developed. Several authors have suggested that microalgae could be used in the production of biodiesel, yet the fuel properties derived from microalgae depend on the saturation and chain length of their fatty acids. Lipid profiles with high ratios of long chains (C${}_{13}$–C${}_{19}$) of saturated and monounsaturated fatty acids have better fuel properties than high ratios of very long chains (≥ C${}_{20}$) (e.g., [24,25,26]). Thraustochytrid species have been considered for biodiesel production as strains that synthesize fatty acids (FAs) have been isolated to produce high-quality fuel [27,28].

It is essential to identify cheap sources of culture media if biodiesel production involving thraustochytrids is to compete with fossil fuels [29]. Modern fisheries use approximately half of their catch for human consumption. Worldwide, about a quarter of the catch, or 20 million tons is discarded as processing waste, byproducts and by-catch [30]. Labyrinthulomycetes are considered saprobes, often found on the dead and decaying macroalgae of leaves where they can excrete extracellular enzymes to chemically alter the detritus for their nutritional benefit [31]. Formulations of culture media from food processing waste may be one way to take advantage of the microbe’s natural ability to obtain nutrients from decaying organic matter. Several authors have examined this potential. Industrial byproducts have been used to formulate culture media for thraustochytrids in the production of omega-3 FAs and biodiesel. Byproducts that have been studied include breadcrumbs [32], soy milk residue [33], stalk juice from sweet sorghum [34], crude glycerol [35,36], and organic waste from breweries [37,38].

The goal of this work was to isolate, identify, and characterize thraustochytrid species from Icelandic coastal samples and to elucidate if and to what extent they would grow in media formulated from fish byproducts.

## 2. Results

### 2.1. Isolation

In total, 39 isolates of presumptive thraustochytrids were obtained in Iceland. All came from three different locations off the north coast of the country: Location A, Skagaströnd (65${}^{{}^{\circ}}$ 49.600′ N 20${}^{{}^{\circ}}$ 18.791, W, degrees decimal minutes), isolates St1, St6, and St7; Location B, Hveravík (65${}^{{}^{\circ}}$ 42.041′ N 21${}^{{}^{\circ}}$ 33.804′ W), isolates St2–St5, St8, and St9; and Location C, Eyjafjörður (65${}^{{}^{\circ}}$ 52.315′ N 18${}^{{}^{\circ}}$ 13.579′ W), isolates St10–St39. Isolates St20, St24, St25, St27–St29, St35, St38, and St39 were not viable (see Table S2). Salinity (34.5 ppt [39]) was similar for all locations while temperature was higher at Location C (30 ${}^{{}^{\circ}}$C [40]) compared to the other two locations (4.5 ${}^{{}^{\circ}}$C [39,41]). Samples from Location C were collected in proximity to a hydrothermal vent along the seafloor.

### 2.2. Phylogeny

**Figure 1:**
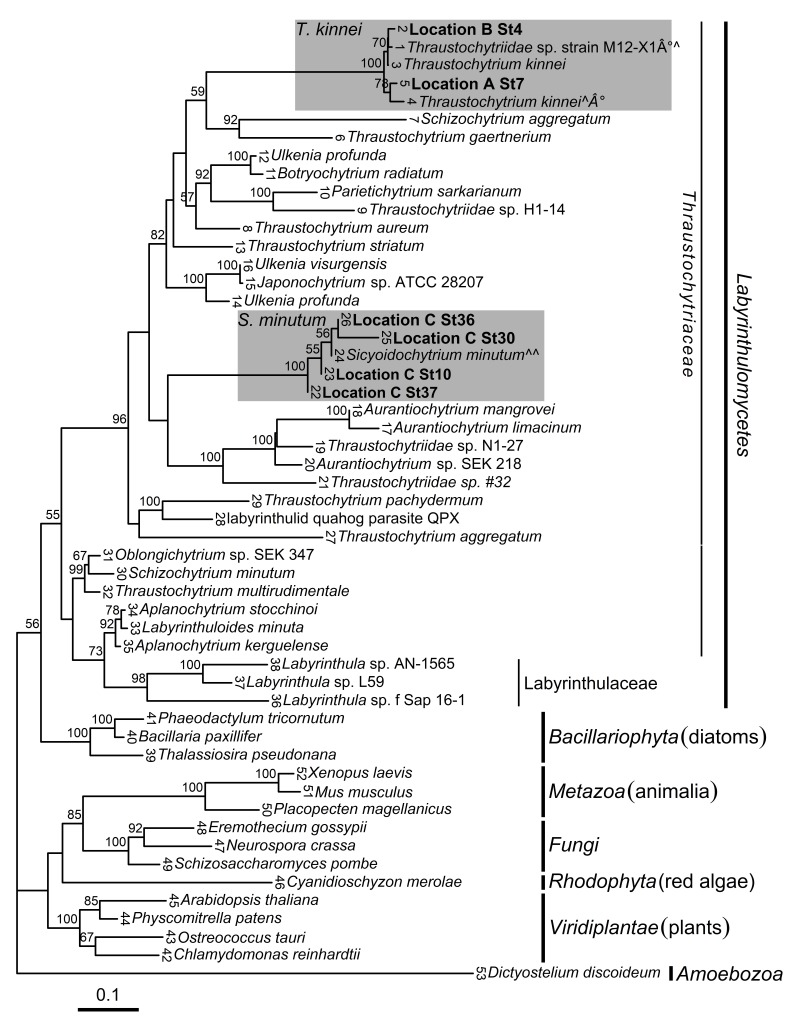
Maximum likelihood phylogenetic tree depicting relationships of Labyrinthulomycetes and other representative eukaryotic species (with amoeba as an out-group) as inferred from partial 18S rRNA gene sequences. Names of taxa are presented as they appear in GenBank. Locations, where new isolates were collected, are indicated in bold (Location A, samples collected from sand and the sea off Skagaströnd; B, Hveravík creek; and Location C, Eyjafjörður fjord). The numbers at each internal branch show bootstrap values (1000 replicates); only values greater than 50% are shown. Leaf numbers are represented in Table S1. Shaded areas indicate clades where new isolates clustered. ${}^{{}^{\circ}}{}^{\wedge }$ denotes Thraustochytriidae sp. strain M12-X1 [42]; ${}^{\wedge }{}^{{}^{\circ}}$*Thraustochytrium kinnei* strain VAL-B1 [43]; and ${}^{\wedge }{}^{\wedge }$ *Sicyoidochytrium minutum* strain SEK 354 [44]. See text for details. Scale bar shows substitutions/site.

**Figure 2:**
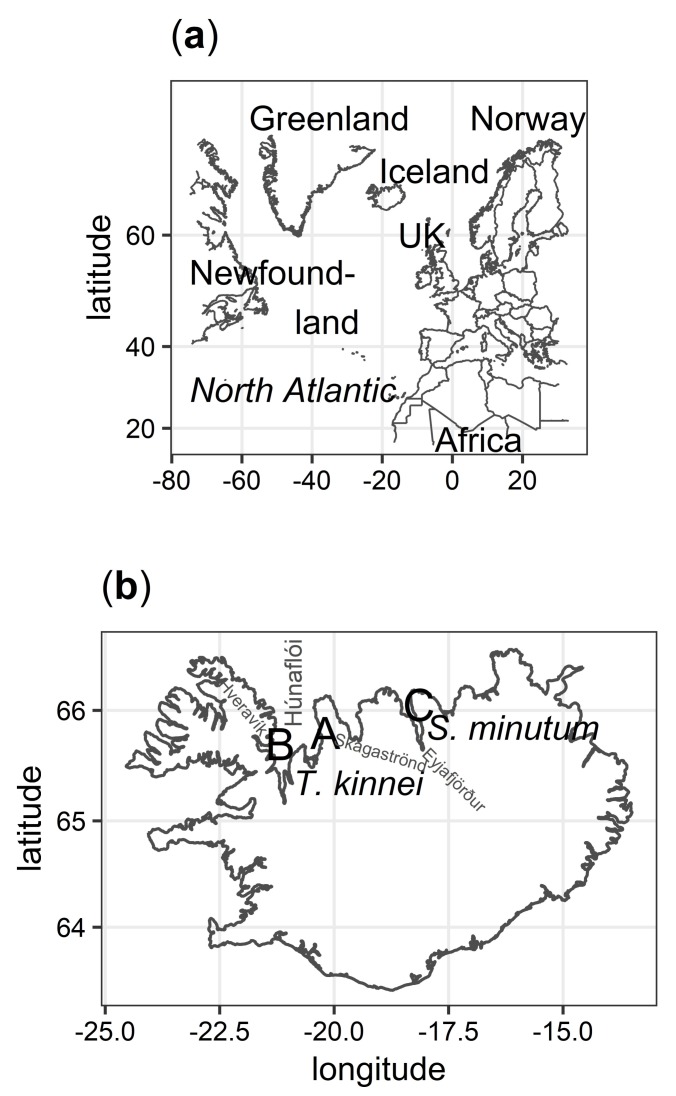
(**a**) A map of the North Atlantic showing Iceland’s location. (**b**) A map of Iceland showing sampling locations of new isolates and species names as they were identified. Location A, samples collected from both sand and sea off the coast of Skagaströnd; Location B, Hveravík creek; and Location C, Eyjafjörður fjord.

Analysis of global sequence diversity showed that new isolates from Locations A and B clustered with *Thraustochytrium kinnei*, while isolates from Location C were affiliated with the *Sicyoidochytrium minutum* species with statistical support (Figure 1 and Figure S1). Locations A and B were located on opposite sides of Húnaflói bay at approximately 60 km distance, while Location C was located in a different fjord (Eyjafjörður) in the north of Iceland (Figure 2). Phylogenetic analysis, with amoeba as an out-group, revealed that the Labyrinthulomycetes had the strongest affiliation with the Stramenopiles (heterokonts).

### 2.3. Biomass and Lipid Production

**Figure 3:**
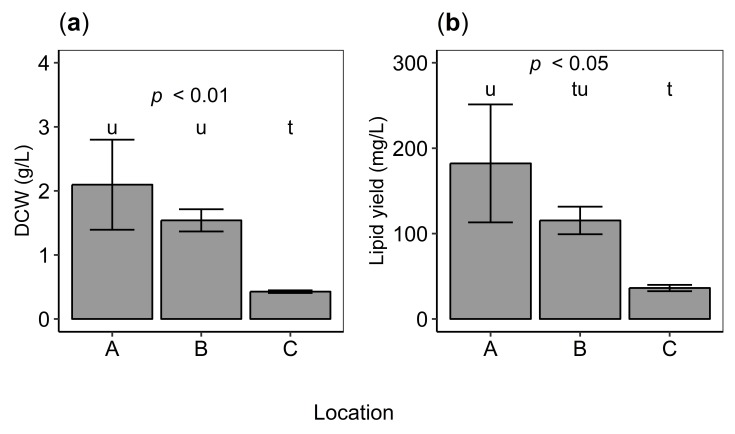
(**a**) Growth (dry cell weight; DCW, g/L) and (**b**) lipid yield (g/L) of isolates from Locations A–C. *P* value indicates statistical significance (analysis of variance, ANOVA). Different letters denote significant differences (Tukey’s HSD test, p<0.01) between locations where the letter “t” shows the lowest value. Error bars indicate standard deviation of the mean of triplicate isolates.

The biomass production of isolates varied between locations, from 0.43±0.02 (C), 1.54±0.17 (Location B) to 2.10±0.70 g/L (Location A) (analysis of variance (ANOVA) [45], $F_{2,6}=12.40,p<0.01$) in GYT media. A post hoc analysis showed that most of the variation was due to the low biomass production of isolates from Location C (Figure 3a).

Lipid yield (mg/L) varied among isolates from different locations (ANOVA, $F_{2,6}=9.52,\;p<0.05$) while lipid content (%) did not (ANOVA, $F_{2,6}=2.22,\;p>0.2$; Figure 3b). Lipid yield was statistically significantly higher for cells from Location A as compared to Location C (Tukey’s HSD test, p<0.05).

#### 2.3.1. Fractional Factorial Analysis

**Figure 4:**
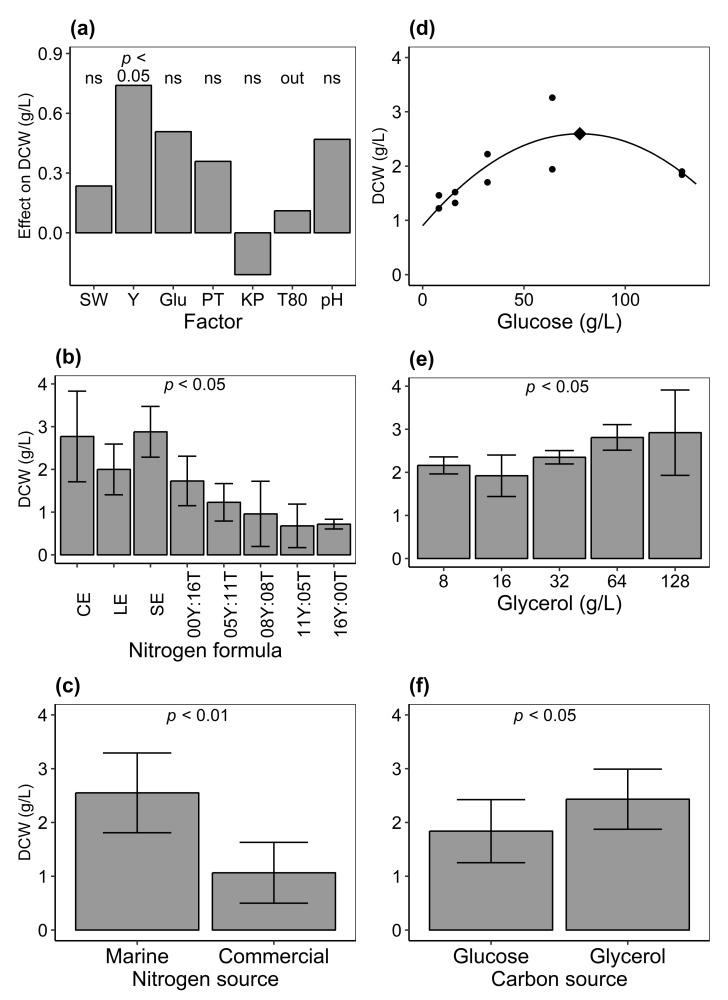
(**a**) Effect of different culture condition factors on the growth (dry cell weight, DCW; g/L) of thraustochytrids in a fractional factorial experiment. See Table 3 and Table 4 for experimental design and Table 1 for the resulting model. Growth (DCW) on various culture media formulations: (**b**) Nitrogen formula consisted of CE, cod; LE, lobster; and SE, shrimp extracts; Y:T, different proportions (g/L) of yeast extract (Y) and tryptone (T). (**c**) Marine or commercial sources of nitrogen. (**d**) Depiction of a quadratic equation where DCW was fitted on glucose concentrations. Diamond shows calculated optimum at 77.6 g/L (see text for equation). (**e**) Different concentrations of glycerol (g/L). (**f**) Glucose or glycerol carbon sources. *P* value indicates statistical significance (analysis of variance (ANOVA) (**b**,**e**) or two-way nested ANOVA (**c**,**f**), see Table 2 for the statistical models); ns, not significant; out, factor not included in the final model. Error bars show the standard deviation of the mean of replicate isolates.

Results from the fractional factorial experimental design on different culture conditions showed that the model that had the best fit included the factors natural seawater (SW), yeast extract (Y), glucose (Glu), peptone (PT), KH${}_{2}$PO${}_{4}$ (KP), and pH. However, Y was the only factor that had a statistically significant effect on growth within the different concentration ranges examined (Table 1 and Figure 4a).

#### 2.3.2. Nitrogen Source

No difference in biomass production was observed between replicate thraustochytrid isolates (ANOVA, $F_{1,14}=1.69,p>0.2$). However, dry cell weight (DCW, g/L) was significantly affected by nitrogen formulae (ANOVA, $F_{7,8}=3.80,p<0.05$, Figure 4b). For different sources of nitrogen, fish protein hydrolysate (2.55±0.74 g/L) had a statistically significantly greater effect on the growth of isolates as compared to commercial nitrogen (1.06±0.57 g/L; Figure 4c and Table 2). This indicates that fish protein hydrolysate from byproducts can be useful nitrogen sources for the cultivation of thraustochytrids.

#### 2.3.3. Carbon Source

Replicate thraustochytrid isolates did not have any statistically significant effect on DCW for either glucose ($F_{1,8}=1.72,p>0.2$) or glycerol ($F_{1,8}=0.80,p>0.3$).

A quadratic model was fitted for DCW (dry cell weight, g/L) on the concentration of glucose (g/L) in culture media (Figure 4d). The equation reads:

$$DCW=-2.81\times 10^{-4}Glu^{2}+4.36\times 10^{-2}Glu+0.90$$

The relationship was statistically significant (linear least-squares model estimate (LM) [46]), multiple $R^{2}=0.64,F_{2,7}=6.22,p<0.05$). In addition, all the coefficients of the equation were statically significant (LM, p<0.05).

DCW varied among glycerol concentrations in media ($F_{1,8}=0.05,p<0.05$; Figure 4e) indicating the effect of the chemical on cell growth. More biomass was obtained when glycerol (2.43±0.56 g/L) was formulated in media as compared to glucose (1.84±0.57 g/L). This indicates that the former was a better carbon source than the latter for growth under current culture conditions (two-way nested ANOVA, $F_{1,8}=5.46,p<0.05$; Figure 4f and Table 2).

### 2.4. Fatty Acid Profile

**Figure 5:**
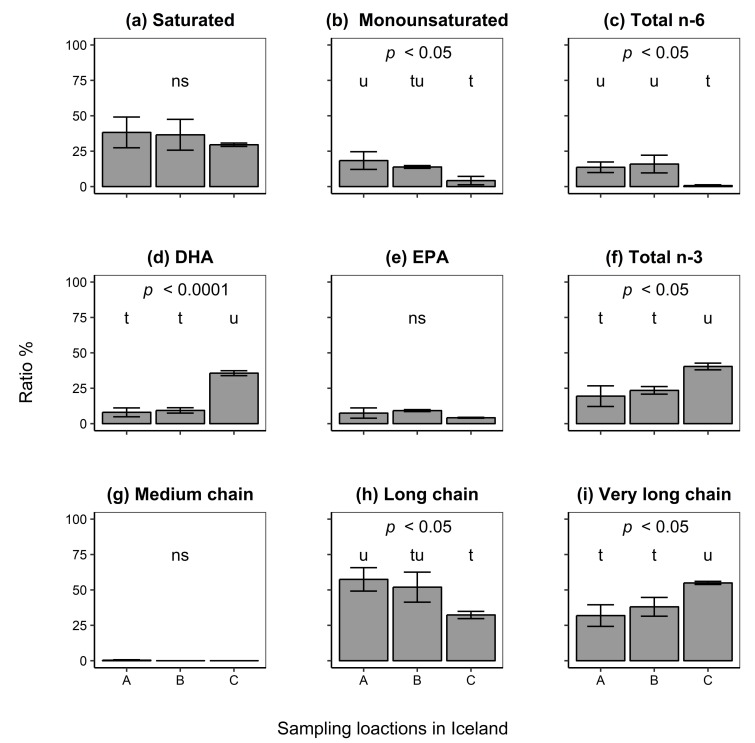
Fatty acid analysis of isolates from three different sampling locations in Iceland. Ratios of the fatty acid types: (**a**) saturated; (**b**) monounsaturated; (**c**) total omega-6; (**d**) docosahexaenoic acid (DHA); (**e**) eicosapentaenoic acid (EPA); and (**f**) total omega-3 are presented, in addition to: (**g**) medium (C${}_{6}$–C${}_{12}$); (**h**) long (C${}_{13}$–C${}_{19}$); and (**i**) very long (≥ C${}_{20}$) fatty acid chain lengths. *P* values indicate statistical significance (analysis of variance, ANOVA). Different letters denote significant differences (Tukey’s HSD test, p<0.05) between locations where the letter “t” shows the lowest value; ns, not significant. Error bars indicate the standard deviation of the mean of triplicate isolates.

Fatty acid profiles were compared among the three different locations current isolates were obtained from (Figure 5). Ratios of FA types were statistically significantly different among locations (multivariate analysis of variance [45], MANOVA${}_{location}$, Wilk’s $\Lambda_{8,6}=8.85\times 10^{-3}$ approximate F=7.22, p<0.05). In addition, further analysis revealed differences in ratios of FA types among locations for monounsaturated (ANOVA, $F_{2,6}=9.60,p<0.05$), total omega-6 (ANOVA, $F_{2,6}=11.43,p<0.01$), docosahexaenoic acid (DHA; ANOVA, $F_{2,6}=133.51,p<0.0001$), and total omega-3 FAs (ANOVA, $F_{2,6}=16.80,p<0.01$). Measurements showed that isolates from Location C (4.21±2.96%) had lower ratios of monounsaturated FAs (Tukey’s HSD test, p<0.05) as compared to Location A (18.38 ± 6.273%), but not when compared to Location B (13.86±0.99%; Tukey’s HSD test, p>0.05). Moreover, isolates from Location C (0.68±0.59%) had low ratios of omega-6 FAs while isolates from Location A (13.64 ± 3.72%) and B (15.91±6.243%) had much higher ratios of the FAs (Tukey’s HSD test, p<0.05). However, cells from Location C (35.65±1.73%) had higher ratios of DHA (Tukey’s HSD test, p<0.0001) when compared to isolates from either Location A (8.00±3.13%) or B (9.34±1.91%). A similar trend was observed for total omega-3 FAs (Tukey’s HSD test, p<0.05) where cells from Location C (40.39±2.38%) had higher ratios of the FAs as compared to those from either Location A (19.43±7.30%) or B (23.52±2.68%).

Differences in FA chain lengths were statistically significant (MANOVA${}_{location}$, Wilk’s $\Lambda_{6,8},$ = 0.021, approximate F=7.91,p<0.01) among isolates from different study sites. Further analysis showed differences in FA chain lengths for isolates among sampling locations for long chain (ANOVA, $F_{2,6}$ = 8.40, p<0.05) and very long chain FAs (ANOVA, $F_{2,6}=12.32,p<0.01$). A post hoc comparison of chain lengths among isolates from different sampling sites revealed that isolates from Location A (57.43±8.27%) had higher ratios of long chain FAs (Tukey’s HSD test, p<0.05) as compared to isolates from Location C (32.31±2.56%). However, these ratios did not differ between isolates from Location B (51.97±10.59%) when compared to isolates from the other two locations (Tukey’s HSD test, p>0.05). Isolates from Location C (54.96±1.10%) had a higher ratio (Tukey’s HSD test, p<0.05) of very long FA chains as compared to those from Location A (31.90±7.66%) and B (38.10±6.64%).

The above results show that variation in both FA types and chain lengths was mostly due to differences between isolates from Location C as compared to those from other locations. This variation indicates the isolates’ diverse culture properties and biotechnological potentials.

### 2.5. Carotenoids

Total carotenoids in cultures of isolates St8 (297.5±27.6μg/g) and St9 (161.0±81.3 μg/g) from Location B were exclusively β carotene.

## 3. Discussion

In this study, two distinct species of Labyrinthulomycetes were isolated from three locations off the northern coast of Iceland: *Thraustochytrium kinnei* and *Sicyoidochytrium minutum*. Although Labyrinthulomycetes have been reported earlier in Iceland [47], the findings presented in this study identify the presence of thraustochytrids off the island’s coast for the first time. Our approach in media formulation showed that thraustochytrids produced more biomass (per unit of substrate) when cultured on nitrogen formulated from hydrolyzed marine fish byproducts as opposed to those cultured on nitrogen of commercial origin. Additionally, glycerol was a better carbon source as compared to glucose in growth experiments. The *S. minutum* species had high ratios of DHA and total omega-3 FAs and low ratios of monounsaturated and omega-6 FAs. These findings point towards the possibility of using *S. minutum* to produce healthy marine oils for consumption and aquaculture. Conversely, the *T. kinnei* isolates from Location A synthesized FA profiles that could be more useful in the production of biodiesel.

### 3.1. Phylogeny

With the advent of nucleic acid sequencing technology, the original classification of Labyrinthulomycetes among the fungi and protozoa [4] was challenged. Current results show that Labyrinthulomycetes classify with the heterokonts. Our results confirm earlier findings that the Labyrinthulomycetes are in fact a monophyletic class of microorganisms that diverge into many genera [44,48,49].

The present analysis shows that the first isolates of thraustochytrids isolated from Icelandic waters belong to the same family but different genera. Strains related to the *T. kinnei* species [5] were isolated in two different locations in the Húnaflói bay. Conversely, isolates from the Eyjafjörður fjord clustered with the *S. minutum* species [44]. The Icelandic isolates of the latter species were collected in proximity to a submarine hydrothermal vent. This may explain their northern presence, as related isolates have been found in warmer waters near the equator [44,50,51]. However, the ecology and speciation of *S. minutum* are not discussed in this paper but could nevertheless warrant further investigation.

While isolates that resembled early descriptions of *T. kinnei* were collected in Kollafjörður (southwest Iceland) in 1974, the earliest reports Labyrinthulomycetes residing in Icelandic waters and sediment were delivered in 1968 [47].

### 3.2. Growth Properties

Overall, isolates from Locations A and B produced more biomass than isolates from Location C. Thraustochytrids produced more biomass per unit of substrate when they were cultured in media containing fish protein hydrolysates as compared to commercial nitrogen sources. This indicates that byproducts from fisheries could be a potential nitrogen source for the formulation for thraustochytrid media. Byproducts from diverse industries have been used to formulate media for thraustochytrids in culture. However, this study introduces the first evidence that byproducts from fisheries may be used in media formulation. Byproducts from food processing [32,34,38,52,53,54] or canteens [55] have been successfully utilized to make culture media for thraustochytrids strains. These results show that byproducts from cod and shrimp processing can easily be used to formulate nitrogen sources for thraustochytrids and could give rise to further research in the field.

When compared to current results, higher biomass production of the related *T. kinnei* strain VAL-B1 (7.2 g/L [43]) was obtained on medium formulated from lupine residue. However, comparable growth performance was observed for another related strain, M12-X1 (2.3 g/L [42]) fermented on beer residue (Figure 1 and Figure S1).

Glycerol was a better carbon source than glucose regarding biomass production. Biomass production of thraustochytrid isolates was higher for glucose-based medium when compared to the strain M12-X1 (1.1 g/L [42]). However, current isolates produced less biomass than previously obtained from strain VAL-B1 (6.5 g/L [43]) fermented on glycerol medium (Figure 1 and Figure S1).

In two of the current isolates (St8 and St9), β-carotene production was found to be higher per unit of biomass (15- and 8-fold, respectively) as compared to its production found in earlier thraustochytrids studies [17]. Additionally, recently developed transgenic strain produced about 30% more pigment as compared to the isolate St8 [56]. These findings indicate a relatively high and naturally occurring ratio of pigment per unit of biomass, which could point towards production potential if culture conditions are further optimized [57].

### 3.3. Fatty Acid Profiles

Higher lipid yield in isolates from Location A as compared to Location C may reflect differences in growth performance as lipid content did not differ among locations. Most variation in FA profiles among the Icelandic isolates stemmed from those that were collected in Location C as compared to those from the other two locations. The results show that ratios of total omega-3 fatty acids were approximately twice as high in isolates from Location C as compared to those from Locations A and B. Ratios of DHA were about four times higher in isolates from Location C as compared to ones from the other two locations. As present analyses show, the isolates from Location C belong to a different species (*S. minutum*) than isolates from Locations A and B (*T. kinnei*), which could explain phenotypic differences. Yokoyama et al. [44] described similar results for another *S. minutum* strain (SEK 354; Figure 1 and Figure S1), indicating that the isolates could be useful in the production in healthy omega-3 fatty acids. Despite being collected at a submarine hydrothermal vent off northern Iceland, the Icelandic *S minutum* isolates have similar phenotypic properties with regard to the FA profiles of related strains from warmer waters [44]. Our results show that the Icelandic *S. minutum* isolates could be a promising candidate for DHA production.

Consumption of PUFAs, in particular DHA and eicosapentaenoic acid (EPA), can reduce the risk of death by stroke and cardiovascular disease [58,59]. Other health benefits of DHA include the maintenance of normal brain function [60] and the prevention of cancer [61].

Omega-3 FAs, especially DHA, are essential components for growth and fish development. Therefore, feeds for farmed fish need substantial amounts of these FAs [62]. Authors have shown that thraustochytrid oil or meal can be adequate alternatives to fish oil for cultured fish (e.g., [63,64,65,66,67]) or molluscs [68,69]. Live thraustochytrids have been used to enrich *Artemia* nauplii and rotifers before they are fed to fish larvae [70,71,72,73]. This practice could be especially important to further develop the hatching of marine fish, as their larvae need live feed throughout the first stages of their life cycle [74].

The *T. kinnei* isolates, which were collected in the north of Iceland, had lower ratios of DHA compared to a related conspecific strain (M12-X1 [42]; Figure 1 and Figure S1). Conversely, the *T. kinnei* isolates from Location A had high ratios of long chain saturated and monounsaturated fatty acids. These FA profiles are considered better biofuels as compared to profiles with high ratios of very long chain FAs due to their oxidative and thermal stability [75,76]. The thraustochytrid strains *Schizochytrium* sp. S056 [28] and *Schizochytrium limacinum* [27] have been considered for biodiesel production due to their FA profiles and rapid growth patterns. The Icelandic *T. kinnei* isolates from Location A had similar FA profiles as these strains, but in order for them to be considered for biofuel production their growth must be further optimized.

## 4. Materials and Methods

### 4.1. Sampling, Isolation, and Culture Maintenance

Samples were collected from 24 sites around Iceland in the summer of 2009 and 2010 (Table S2). The samples (various seaweed, sand, small stones, and seawater) were put into sterile bottles. The samples were brought chilled (4 ${}^{{}^{\circ}}$C) to the laboratory where the material was handled in three different ways: seaweed and stones rinsed with sterile 30% natural seawater (SW) to make up an incubation broth (2 g/L glucose, 1 g/L yeast extract, 2 g/L peptone (all from BD Difco), 0.3 g/L penicillin and 0.5 g/L streptomycin); seaweed and stones were put into sterile SW (either 30% or 100%) and baited with pine pollen; or pine pollen bait was put directly into samples containing seaweed and sand. The samples were incubated for up to 15 days at 22 ${}^{{}^{\circ}}$C, and then were moved to GYP agar plates (containing incubation broth in addition to 14 g/L agar and 60% SW at 25 ${}^{{}^{\circ}}$C). Colonies showing thraustochytrid characteristics in stereo- or microscopes were repeatedly transferred to new agar plates until pure isolates were obtained. Cultures of presumptive thraustochytrids were maintained either on GYP agar plates (without antibiotics) or kept frozen at −80 ${}^{{}^{\circ}}$C in GYP broth (GYP agar medium without the agar) containing 12% or 20% glycerol.

### 4.2. Growth Studies

An experiment was carried out to examine growth and lipid production of isolates (in triplicates) from Locations A (isolates St1, St6, and St7), B (St4, St5, and St8), and C (St30, St31, and St32). Colonies from GYP agar plates were used to inoculate 10 mL of GYT medium (5% glucose, 4 g/L yeast extract, 4 g/L tryptone, 0.2 g/L KH${}_{2}$PO${}_{4}$ (all from BD Difco) and 30% natural seawater; they were grown at 25 ${}^{{}^{\circ}}$C while shaking at 150 rpm) in 50 mL Erlenmeyer flasks and cultured for three days. Subsequently, the cultures were transferred to 50 mL GYT medium in 250 mL Erlenmeyer flasks and incubated for seven days. Cells were spun down (6000× *g* for 10 min), washed twice with 30 mL sterile water, dried in an incubator at 105 ${}^{{}^{\circ}}$C for 16 h and weighed. Biomass was determined with dry cell weight (DCW, g/L).

A fractional factorial experimental design [77] was used to study the effect that different culture conditions had on biomass production (Table 3 and Table 4) of isolate St5.

The influence of marine-derived nitrogen on growth was studied on duplicate isolates (St11 and St17) by formulating various media. The marine nitrogen sources used were cod extract (CE), lobster extract (LE) and shrimp extract (SE), which were all obtained from SERO ltd. (Skagaströnd, Iceland). They were produced by enzymatic hydrolysis of fish byproducts. Media were formulated using 16 g/L of each extract. For comparison to commercially derived nitrogen, yeast extract (Y) and tryptone (T) (both from BD Difco) were used. These were formulated in different proportions: 00Y:16T, 05Y:11T, 08Y:08T, 11Y:05T, and 16Y:00T g/L, bringing the total number of nitrogen formulae examined up to eight. Growth experiments were carried out as described above.

To examine the effect of carbon source on growth, media were formulated with different concentrations of glucose and glycerol (8, 16, 32, 64 and 128 g/L) and growth experiments carried out (on isolates St11 and St17) with a commercial nitrogen source as above for GYT medium.

The effect of culture condition factors on growth was analyzed using the stepwise selection algorithm (both directions) based on the Akaike Information Criteria (AIC) as a quality criterion [78]. AIC selects factors that explain variation in DCW, and the model with the best fit was selected as the final model. Analysis of variance (ANOVA [45]) was used to examine the effect of duplicate thraustochytrid isolates on growth performance (DCW). The effect of different nitrogen formulae and glucose concentrations on the growth was analyzed using ANOVA. To examine the effect that either the source of nitrogen or carbon had on growth, an analysis of two-way nested ANOVA was carried out where nitrogen formulae were nested within in the former and chemical concentrations within the latter source. A quadratic model was fitted for DCW on the concentration of glucose in culture media with a linear least-squares model estimate (LM [46]).

Growth data were presented as mean values of DCW and the standard deviation of the mean (SD). Statistical analysis was carried out in R [79].

### 4.3. Fatty Acid Analysis

Fatty acid profiles of new isolates were analyzed from dry cells (three replicates from Location A (St1, St6, and St7), Location B (St4, St5, and St8), and Location C (St30, St31, and St32) cultured in GYT media as above) using the Bligh and Dyer method [80]. Fatty acid methyl esters (FAMEs) were synthesized according to the AOCS Official Method Ce 1b-89 with minor changes. FAMEs were separated on a Varian 3900 gas chromatography equipped with a fused silica capillary column (HP-88, 100 m × 0.25 mm × 0.20 μm film), split injector and flame ionisation detector based on the AOAC 996.06 method. Data were collected with the Galaxie Chromatography Data System, Version 1.9.3.2 software. The fatty acid analysis was carried out at Matís o.hf., Reykjavík.

To examine the effect that the location factor had on the ratios of FAs or chain lengths, multivariate analysis of variance, (MANOVA, Wilk’s test [45]) was carried out across FA types on one hand and chain length on the other. Similarly, to determine the effect that location had on the ratios of each FA type (saturated; monounsaturated; total omega-6; docosahexaenoic acid (DHA); eicosapentaenoic acid (EPA); total omega-3) or chain length (short: C${}_{2}$–C${}_{5}$; medium: C${}_{6}$–C${}_{12}$; long: C${}_{13}$–C${}_{19}$; and very long: ≥ C${}_{20}$), ANOVA was carried out on each type or chain length. When statistically significant differences were detected (*P*< 0.05), a post hoc analysis was carried out, employing Tukey’s honest significant difference (HSD) test for multiple comparisons among locations [45]. Fatty acid data were presented as mean ratios (%) and SD of the mean. Statistical analysis was carried out in R [79].

### 4.4. Carotenoids

A total of 50 mg of whole cells from the isolates St8 and St9 (two replicates each, cultured in GYT medium as above) were harvested as above and carotenoids eluted in acetone. Separation of carotenoids was carried out by high-performance liquid chromatography (HPLC) on a silica gel column (Supelco LiChrospher SI-60, 250 × 4.6 mm, 5 μm) using acetone:hexane (18:82, v/v) as the mobile phase at a flow rate of 1.2 mL/min with detector set at 474 nm. To determine the total carotenoid content, 50 mg of whole cells (see above) from two replicates of the isolates St8 and St9 from Location B (cultured in GYT medium as above) were placed in 100 mL of acetone (containing 500 mg/L butylated hydroxytoluene; BHT) and absorbance measured at 474 nm.

### 4.5. DNA Sequencing

For the sequencing reactions of the 18S rRNA gene, DNA was harvested from monocultures cultured in GYT medium (see above). DNA was extracted using the phenol-chloroform method [81]. The DNA was PCR amplified using *Teg* polymerase (Matís ohf., Reykjavík Iceland; denaturation at 94 ${}^{{}^{\circ}}$C for 5 min followed by 30 cycles of denaturation at 94 ${}^{{}^{\circ}}$C for 45 s, annealing at 62 ${}^{{}^{\circ}}$C for 30 s and extension at 72 ${}^{{}^{\circ}}$C for 90 s with a final extension at 72 ${}^{{}^{\circ}}$C for 10 min) using 18S primers: 18S001F forward and 18S13R reverse [6,82]. The product was cleaned using QIAquick PCR Purification Kit (Qiagen) and the sequencing carried out on ABI 3730XL capillary sequencer at Macrogen Inc. (Amsterdam, The Netherlands).

### 4.6. Phylogeny

The 18S rDNA sequences were aligned and compared to known sequences in the GenBank database of the National Center for Biotechnology Information [83] using the Basic Local Alignment Search Tool (BLAST [84]). Sequences of the new isolates in addition to the ones retrieved from GenBank (Table S1) were used for phylogenetic analysis. The sequences were aligned using MAFFT with the L-INS-i strategy [85]. The robustness of 25 different nucleotide substitution models were each evaluated with goodness-of-fit and measured by the Bayesian information criterion (BIC [86,87]) and the model with the lowest BIC scores were used. The evolutionary history was inferred by using the maximum likelihood method (ML [88]) based on the Tamura–Nei model (TN93 [89]). A discrete gamma (+G) distribution was used to model evolutionary rate differences among sites (4 categories). The trees were drawn to scale, with branch lengths measured in the number of substitutions per site. Phylogenetic trees were implemented in MEGA X [90] with bootstrap values calculated with 1000 replications.

### 4.7. Nucleotide Data Accession Numbers

The partial sequences of the 18S rRNA gene for isolates were deposited into NCBI’s GenBank repository under the accession numbers KX430103 (St4), KX430104 (St7), KX430105 (St10), KX430106 (St30), KX430107 (St36), and KX430108 (St37).

If not otherwise stated, all chemicals were obtained from Sigma-Aldrich Co. LLC. (USA).

## 5. Conclusions

In this study, we identified the first thraustochytrids isolates collected off the coast of Iceland. They were collected from three sites off the country’s northern coast and belong to two different genera, which may explain phenotypic variation. The *T. kinnei* isolates that were sampled from Location A (Skagaströnd) are possible candidates for biodiesel production, as they synthesized high ratios of saturated and monounsaturated long chain FAs. The isolates collected from Location C (Eyjafjörður) were identified as belonging to the *S. minutum* species, and contain low ratios of monounsaturated and omega-6 fatty acids and high ratios of DHA omega-3 FAs. This renders the isolates suitable for the production of marine oils for human consumption or to supplement aquaculture feeds. While *T. kinnei* exhibited a better growth performance than *S. minutum*, isolates of the latter species grew better in media formulated from glycerol as compared to glucose. Thraustochytrids cultivated in media containing fish protein hydrolysates produced more biomass per unit of substrate when compared to conspecifics that were grown on commercial nitrogen sources. This is the first study to show that media formulated from fishery byproducts can be effective for biomass production in thraustochytrids, and thereby necessitates further investigation. The Icelandic *S. minutum* isolates were collected in the proximity to a submarine hydrothermal vent. Current results show that they were related to conspecifics that were obtained closer to the equator, and that FA profiles were similar among these geographically distant isolates. These findings could give rise to further research into the ecology and speciation of thraustochytrids. 

## Figures and Tables

**Table 1:** Culture condition factors that explain variation in growth (dry cell weight; DCW, g/L) of thraustochytrids as analysed with stepwise model selection (both directions). The model with the best fit is shown.

Factor	Acronym	Unit		Df ${}^{a}$	Sum Sq	Mean Sq	*F* value	p−value ${}^{b}$
Natural seawater	SW	% vs. dH${}_{2}$O		1	0.1659	0.1659	1.227	0.3184
Glucose	Glu	g/L		1	0.7737	0.7737	5.723	0.0622
Yeast extract	Y	g/L		1	1.6435	1.6435	12.157	0.0175
Peptone	PT	g/L		1	0.3863	0.3863	2.857	0.1518
KH${}_{2}$PO${}_{4}$	KP	g/L		1	0.1321	0.1321	0.977	0.3683
Starting pH- value	pH	pH		1	0.6613	0.6613	4.891	0.0779
Residuals				5	0.676	0.1352		

${}^{a}$ Df: Degrees of freedom. ${}^{b}$ Probability of having more extreme variance component and *F*-statistic than the observed values by chance alone.

**Table 2:** Two-way nested analysis of variance on the effect of media sources on the growth (dry cell weight (DCW), g/L) of thraustochytrids.

Nitrogen	Df ${}^{e}$	Sum Sq	Mean Sq	*F* value	p−value ${}^{f}$
Nitrogen source ${}^{a}$	1	8.281	8.281	20.610	0.0019
Nitrogen formula ${}^{b}$	6	2.415	0.403	1.002	0.4843
Residuals	8	3.214	0.402		
**Carbon**					
Carbon source ${}^{c}$	1	1.764	1.7642	7.291	0.0223
Concentration ${}^{d}$	8	3.486	0.4358	1.801	0.1891
Residuals	10	2.42	0.242		

${}^{a}$ Origin of nitrogen source: marine and commercial. ${}^{b}$ Nitrogen formula: Nitrogen was formulated using three sources of marine origin and in five different proportions of yeast extract and tryptone (commercial origin). Nitrogen formulae were nested within nitrogen sources. ${}^{c}$ Carbon source: glucose and glycerol. Chemical concentrations were nested within carbon source. ${}^{d}$ Concentration of carbon source in media. ^e^ Df: Degrees of freedom. ${}^{f}$ Probability of having more extreme variance component and *F*-statistic than the observed values by chance alone.

**Table 3:** Growth of thraustochytrids: Culture condition factors and low and high values in a fractional factorial experimental design.

Factor	Low	High
Natural seawater	10	40
Glucose	10	50
Yeast extract	3	8
Peptone	3	8
KH${}_{2}$PO${}_{4}$	0	0.1
Tween 80 ${}^{a}$	0	0.1
Starting pH-value	6	8

${}^{a}$ Unit, g/L; see Table 1 for units on other factors.

**Table 4:** Growth of thraustochytrids: Fractional factorial experimental design of culture conditions.

Run	SW ${}^{a}$	Glu	Y	PT	KP	NH${}_{4}$
1	1	−1	1	−1	−1	1
2	1	1	−1	1	−1	−1
3	−1	1	1	−1	1	−1
4	1	−1	1	1	−1	−1
5	1	1	−1	1	1	1
6	1	1	1	−1	1	−1
7	−1	1	1	1	−1	1
8	−1	−1	1	1	1	1
9	−1	−1	−1	1	1	−1
10	1	−1	−1	−1	1	1
11	−1	1	−1	−1	−1	1
12	−1	−1	−1	−1	−1	−1

${}^{a}$ See Table 3 for low (−1) and high (1) values of culture condition factors and Table 1 for abbreviations.

## Supplementary Materials

The following are available online at https://www.mdpi.com/1660-3397/17/8/449/s1, Figure S1: Maximum likelihood phylogenetic tree depicting relationships of Labyrinthulomycetes species as inferred from partial 18S rRNA gene sequences, Figure S2: Detailed fatty acid profile of all viable isolates of the ones originally collected off the coast of Iceland, Table S1: Taxa and accession numbers represented in this study and for which analyses corresponding sequences were used, Table S2: Locations and materials sampled for the isolation of thraustochytrids in 2009 and 2010.
