# Supplementary material for: Isolation, Characterization and Biotechnological Potentials of Thraustochytrids from Icelandic Waters
Source: Mar Drugs. 2019 Jul 31;17(8):449. doi: 10.3390/md17080449 (PMC6723786; doi:10.3390/md17080449)
Supplement: Supplementary file 1 [file marinedrugs-17-00449-s001.pdf]

Supplementary Materials: Isolation, Characterization and Biotechnological Potentials of Thraustochytrids from Icelandic Waters

Magnús Örn Stefánsson [1,2,†,*], Sigurður Baldursson [1,2†], Kristinn P. Magnússon [1,3], Arnheiður Eyþórsdóttir [1] and Hjörleifur Einarsson [1]

Electronic Supplementary Material

Taxa and GenBank accession numbers of sequences, that were used in current study can be viewed in Table S1. The table also shows node numbers of reconstructed phylogenetic trees.

Location and material sampled for the isolation of thraustochytrid strains in Iceland are shown in Table S2.

The evolutionary history of selected Labyrinthulomycetes isolates as inferred on the basis of 18S rRNA gene sequence variation among strains is shown in Figure S1.

Detailed fatty acid profiles for all viable isolates of the 39 collected are show in Figure S2.

References

1. Quilodrán, B.; Hinzpeter, I.; Quiroz, A.; Shene, C. Evaluation of liquid residues from beer and potato processing for the production of docosahexaenoic acid (C22:6n-3, DHA) by native thraustochytrid strains. *World J. Microbiol. Biotechnol.* **2009**, *25*, 2121–2128. doi:10.1007/s11274-009-0115-2.
2. Silva, D.; Villarroel, M.P.; Roa, A.L.; Quilodrán, B.H. Use of waste from agroindustrial sources as substrate for polyunsaturated fatty acids production by *Thraustochytrium kinney* VAL-B1. *Int. J. Eng. Res. Afr.* **2017**, *33*, 50–55. doi:10.4028/www.scientific.net/JERA.33.50.
3. Yokoyama, R.; Salleh, B.; Honda, D. Taxonomic rearrangement of the genus *Ulkenia* sensu lato based on morphology, chemotaxonomical characteristics, and 18S rRNA gene phylogeny (Thraustochytriaceae, Labyrinthulomycetes): emendation for *Ulkenia* and erection of *Botryochytrium*, *Parietichytrium*, and *Sicyoidochytrium* gen. nov. *Mycoscience* **2007**, *48*, 329–341. doi:10.1007/s10267-007-0377-1.

Supplementary Table S1. Taxa and accession numbers represented in this study and for which analyses corresponding sequences were used.

Taxon[a]	Accession number[b]	Node number[c]	
		E	L
Stramenopiles (heterokonts)			
Labyrinthulomycetes			
Thraustochytriaceae			
Aplanochytrium kerguelense	AB022103	35	52
Aplanochytrium stocchinoi	AJ519935	34	50
Aurantiochytrium limacinum	AB022107	17	25
Aurantiochytrium mangrovei	DQ367049	18	26
Aurantiochytrium sp. SEK 218	AB290573	20	27
Botryochytrium radiatum	AB022115	11	18
Japonochytrium sp. ATCC 28207	AB022104	15	23
labyrinthulid quahog parasite QPX	AY052644	28	46
Labyrinthuloides minuta	L27634	33	51
Oblongichytrium sp. SEK 347	AB290575	31	48
Parietichytrium sarkarianum	AB355411	10	20
Schizochytrium aggregatum	AB022106	7	15
Schizochytrium minutum	AB022108	30	47
Sicyoidochytrium minutum	AB355412	24	38
Sicyoidochytrium sp. SEK 675	AB973513		30
Sicyoidochytrium sp. SEK 676	AB973514		34
Thraustochytriidae sp. #32	DQ367052	21	29
Thraustochytriidae sp. Fng1	AY870336		37
Thraustochytriidae sp. H1-14	AB073305	9	19
Thraustochytriidae sp. MBIC11060	AB183653		32
Thraustochytriidae sp. MBIC11063	AB183654		31
Thraustochytriidae sp. MBIC11071	AB290585		35
Thraustochytriidae sp. MBIC11077	AB183659		40
Thraustochytriidae sp. MBIC11078	AB290582		36
Thraustochytriidae sp. N1-27	AB073308	19	28
Thraustochytriidae sp. NK40	KM233917		33
Thraustochytriidae sp. strain M12-X1	DQ459552	1	5
Thraustochytrium aff. *kinnei* BAFCcult 3485	HQ228962		11
Thraustochytrium aff. *kinnei* BAFCcult 3489	HQ228963		10
Thraustochytrium aff. *kinnei* BAFCcult 3490	HQ228964		12
Thraustochytrium aff. *kinnei* BAFCcult 3495	HQ228966		6
Thraustochytrium aff. *kinnei* BAFCcult 3497	HQ228967		7
Thraustochytrium aggregatum	AB022109	27	44
Thraustochytrium aureum	AB022110	8	16
Thraustochytrium gaertnerium	AY705753	6	14
Thraustochytrium kinnei	DQ367053	3	4
Thraustochytrium kinnei	KF460462		2

cont. on next page

Supplementary Table S1 *cont.*

Taxon[a]	Accession number[b]	Node number[c]	
		E	L
Stramenopiles (heterokonts)			
Labyrinthulomycetes			
Thraustochytriaceae			
Thraustochytrium kinnei	KF460466		1
Thraustochytrium kinnei	KF709393	4	9
Thraustochytrium kinnei	L34668		13
Thraustochytrium multirudimentale	AB022111	32	49
Thraustochytrium pachydermum	AB022113	29	45
Thraustochytrium striatum	AB022112	13	21
Ulkenia profunda	AB022114	12	17
Ulkenia profunda	L34054	14	22
Ulkenia visurgensis	AB022116	16	24
Labyrinthulaceae			
Labyrinthula sp. AN-1565	AB022105	38	54
Labyrinthula sp. f Sap 16-1	AF348522	36	53
Labyrinthula sp. L59	AB095092	37	55
Bacillariophyta (diatoms)			
Bacillaria paxillifer	M87325	40	58
Phaeodactylum tricornutum	GQ452861	41	57
Thalassiosira pseudonana	HM991698	39	56
Viridiplantae (plants)			
Arabidopsis thaliana	GQ380689	45	
Chlamydomonas reinhardtii	JX888472	42	
Ostreococcus tauri	GQ426346	43	
Physcomitrella patens	AF223015	44	
Rhodophyta (red algae)			
Cyanidioschyzon merolae	AF441376	46	
Fungi			
Eremothecium gossypii	AY046265	48	
Neurospora crassa	AY046271	47	
Schizosaccharomyces pombe	EU011742	49	
Metazoa (animals)			
Mus musculus	X00686	51	
Placopecten magellanicus	X53899	50	
Xenopus laevis	X04025	52	
Amoebozoa			
Dictyostelium discoideum	KJ394480	53	59

[a]Names of taxa are presented as they appear in GenBank

[b]GenBank accsession numbers of 18S rRNA gene

[c]Leaf number of sequences used to reconstruct phylogenetic trees of E, Eukaryotes; L, Labyrinthulomycetes.

Supplementary Table S2. Locations (degrees decimal minutes) and materials sampled for the isolation of thraustochytrids in 2009 and 2010.

**Location (symbol)**	**Lat**	**Lon**	**Material**[a]	**Isolate**
Spákonufellshöfði, Skagaströnd (A)	65° 50.073' N	20° 18.954' W	SWD and SW	St1, St6, St7
Hveravík south of Drangsnes (B)	65° 42.041' N	21° 33.804' W	SW	St2 – St5, St8, St9
Hverastrýtur, Eyjafjörður (C)	65° 52.315' N	18° 13.579' W	Stones and SWD	St10 – St39[b]

[a]SW, seawater; SWD, seaweed

[b]Isolates St20, St24, St25, St27 - St29, St35, St38, and St39 were not viable in culture subsequent to isolation

Further sampling effort was made at other locations around Iceland which did not result viable isolates. These are listed below. Skagaströnd: 65° 49.600' N, 20° 18.791' W, Sand and SW; By Hrafná Skagaströnd: 65° 49.078' N, 20° 18.636' W, SWD and SW; By Hólanes Skagaströnd: 65° 49.342' N, 20° 18.517' W, SWD and SW; Reykjanes by Ísafjarðardjúp: 65° 55.633' N, 22°25.300' W, SWD and SW; Grindavík: 63° 51.083' N, 22° 21.372' W, SWD and SW; Hafnir: 63° 56.395' N, 22° 37.883' W, SWD and SW; Húnaflói (3 samples): 65° 51.566' N, 20° 53.599' W, SW; Gjögur: 65° 58.902' N, 21° 20.71' W, SW; Djúpavík: 65° 57.930' N, 21° 32.92' W, SW; Sölvabakki: 65° 42.79' N , 20° 17.97' W, SW; Stapi: 65° 47.36' N , 20° 18.63' W, SW; Gjögur: 65° 58.902' N, 21° 20.71' W, SW; Djúpavík: 65° 57.93' N, 21° 32.92' W, SW; Stapi: 65° 47.36' N, 20° 18.63' W, SW; Sölvabakki: 65° 42.79' N, 20° 17.97' W, SW; By Hrafná: 65° 49.078' N, 20° 18.636' W, SW; Hallá: 65° 47.211' N, 20° 17.940' W, SW; Stapi: 65° 47.36' N, 20° 18.63' W, SW; Sölvabakki: 65° 42.79' N, 20° 17.97' W, SW; Laxárvík: 65° 43.67' N, 20° 16.323' W, SW; Hafurstaðaá: 65° 46.766' N, 20° 16.765' W, SW.

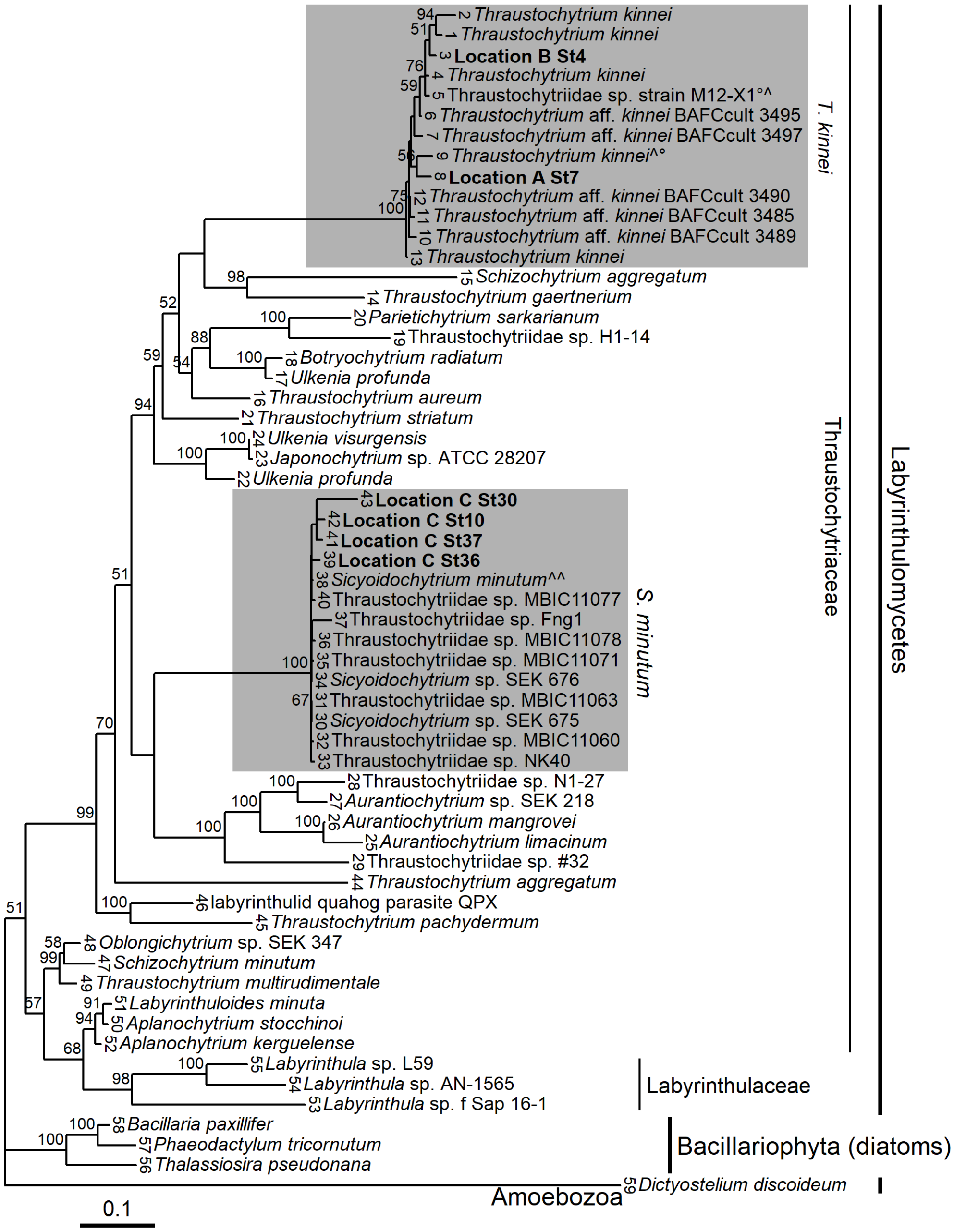

Figure S1. Maximum likelihood phylogenetic tree depicting relationships of Labyrinthulomycetes species (with amoeba as an outgroup) as inferred from partial 18S rRNA gene sequences. Names of taxa are presented as they appear in GenBank. Locations where newly isolated strains were collected are indicated in bold (location A, samples collected from sand and the sea off Skagastönd; B, Hveravík creek; and C, Eyjafjörður fjord). Shaded areas indicate clades where newly isolated strains cluster including related strains. The numbers at each internal branch show bootstrap values (1000 replicates); only values greater than 50% are shown. °^ denotes Thraustochytriidae sp. strain M12-X1 [1]; ^° *Thraustochytrium kinnei* strain VAL-B1 [2]; and ^^ *Sicyoidochytrium minutum* strain SEK 354 [3] see text for details. Scale bar shows substitutions/site.

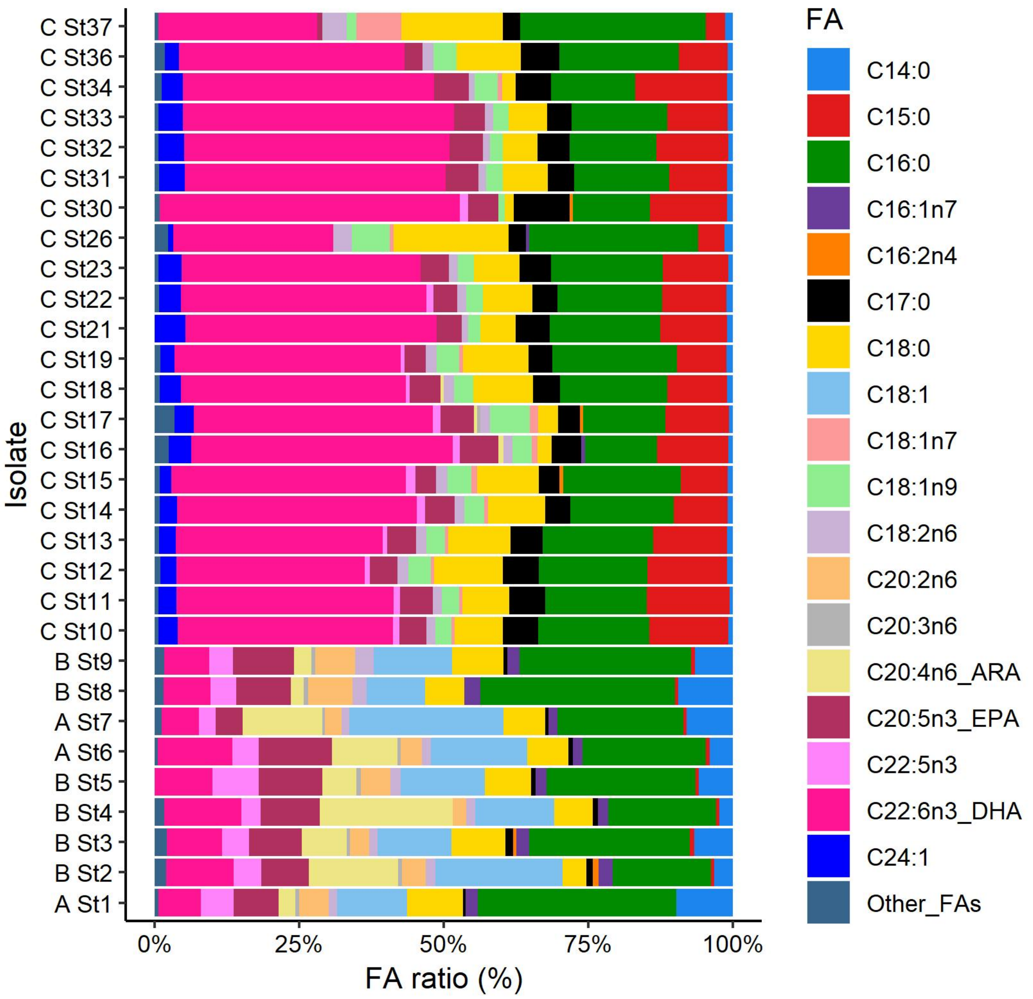

Figure S2. Detailed fatty acid (FA) profile of all viable isolates of the ones originally collected off the coast of Iceland (capital letters denote location: A, Skagaströnd; B, Hveravík; and C, Eyjafjörður). Fatty acids constituting more than 1.5% of total ratio are shown. FAs are ordered according to chain length with arachidonic acid (ARA), eicosapentaenoic acid (EPA), and docosahexaenoic acid (DHA) named especially.
